# PET-based risk stratification for adverse events under [^225^Ac]Ac-PSMA radioligand therapy

**DOI:** 10.1007/s00259-026-07788-4

**Published:** 2026-02-09

**Authors:** Liam Widjaja, Sophie C. Siegmund, Franz J. Gildehaus, Nina-Sophie Schmidt-Hegemann, Martin G. Pomper, Steven P. Rowe, Ralph A. Bundschuh, Vera Wenter, Gabriel T. Sheikh, Konrad Klimek, Christian G. Stief, Mathias J. Zacherl, Rudolf A. Werner, Jozefina Casuscelli

**Affiliations:** 1 Department of Nuclear Medicine, LMU University Hospital, LMU Munich, Marchioninistr. 15 , D-81377 Munich, Germany; 2 Bavarian Cancer Research Center (BZKF), partner site Munich, Munich, Germany; 3 Department of Radiation Oncology, LMU University Hospital, LMU Munich, Munich, Germany; 4 Department of Radiology, University of Texas Southwestern Medical Center, Dallas, TX USA; 5 Department of Nuclear Medicine, University Hospital Carl Gustav Carus Dresden, Dresden, Germany; 6 Institute of Radiopharmaceutical Cancer Research, Helmholtz Zentrum Dresden- Rossendorf (HZDR), Rossendorf, Germany; 7 German Cancer Consortium (DKTK), Partner Site Dresden, Dresden, Germany; 8 Faculty of Medicine, National Center for Tumor Diseases (NCT), NCT/UCC Dresden, University Hospital Carl Gustav Carus, TUD Dresden University of Technology, Helmholtz-Zentrum Dresden-Rossendorf (HZDR), Dresden, Germany; 9 Department of Urology, LMU University Hospital, LMU Munich, Munich, Germany; 10 German Cancer Consortium (DKTK), partner site Munich, a partnership between DKFZ and LMU University Hospital Munich, Munich, Germany

**Keywords:** Prostate carcinoma, PSMA, Radioligand therapy, PSMA-PET, [^225^Ac]Ac-PSMA RLT, Side effects

## Abstract

**Purpose:**

Actinium-225 prostate-specific membrane antigen radioligand therapy ([^225^Ac]Ac-PSMA RLT) is a promising salvage option for metastatic castration-resistant prostate cancer (mCRPC). This study aimed to identify predictive parameters for renal and hematological adverse events.

**Methods:**

Twenty-six patients undergoing [^225^Ac]Ac-PSMA-I&T RLT (mean 3 ± 1 cycles) were retrospectively included. All patients received pretherapeutic Fluor-18 ([^18^F]F)-PSMA-1007 positron emission tomography/computed tomography (PET/CT) to quantify renal standardized uptake values (SUV) through manual segmentation of both kidneys. Total and osseous PSMA-tumor volume (TV) were also calculated. Additionally, Technetium-99m ([^99m^Tc]Tc)-mercaptoacetyltriglycine (MAG3) renal scintigraphy was performed to assess tubular extraction rates (TER). Standard laboratory values were evaluated at baseline, at and between treatment cycles, including estimated glomerular filtration rate (eGFR) and hemoglobin (Hb). Renal events were defined as an eGFR decline ≥ 20% (*Kidney Disease: Improving Global Outcomes (KDIGO) CKD Work Group* recommendations), while anemia was classified according to common terminology criteria of adverse events (CTCAE) version 5.

**Results:**

Both eGFR (*P* = 0.0018) and Hb (*P* = 0.0002) declined during therapy. Eight patients (31%) experienced renal events, and ten subjects (38%) developed grade 3 anemia. Renal SUV on [^18^F]F-PSMA-1007 PET/CT correlated with both eGFR and TER (*r* ≥ 0.413, *P* ≤ 0.036), suggesting that PET-based renal quantification may reflect renal function. SUV emerged as the strongest predictor of renal events (Odds Ratio [OR] = 0.095, *P* = 0.011) outperforming TER (*P* = 0.165) and baseline eGFR (*P* = 0.245). Based on Kaplan Meier analysis, low SUV was also associated with shorter renal event-free survival (*P* = 0.001). Baseline Hb (*P* = 0.002) was the strongest predictor of grade 3 anemia, while additional markers (platelets, white blood cell count, alkaline phosphatase, osseous PSMA-TV and lactate dehydrogenase; each *P* ≤ 0.037) also contributed. In line, baseline Hb emerged as the strongest predictor of anemia-free survival (*P* = 0.0007; osseous TV, *P* = 0.024).

**Conclusion:**

PET-derived renal SUV and osseous tumor volume are promising predictive markers for renal and hematological adverse events during [^225^Ac]Ac-PSMA-I&T RLT. These findings may support pretherapeutic risk stratification in this vulnerable patient population.

## Introduction

Metastatic castration-resistant prostate cancer (mCRPC) remains a therapeutic challenge, particularly in patients progressing after multiple lines of therapy [1]. Prostate-specific membrane antigen (PSMA)-targeted radioligand therapy (RLT) with the α-emitter Actinium-225 ([^225^Ac]Ac-PSMA RLT) has emerged as an effective salvage option for patients with metastatic castration-resistant prostate cancer (mCRPC) [2]. Due to its high linear energy transfer (LET), Actinium-225 induces intense and localized DNA damage, producing therapeutic responses even in patients who have progressed following β⁻-emitter Lutetium-177 ([¹⁷⁷Lu]Lu)-PSMA RLT [3]. A 2024 published multicenter trial investigating [²²⁵Ac]Ac-PSMA RLT reported on a median progression-free survival of 7.9 months [4]. Furthermore, a review including 18 studies with 1,155 patients demonstrated a ≥ 50% decline of prostate specific antigen (PSA) level in 65% of the cases [2]. However, most studies were conducted monocentric and retrospectively, thus for final evidence prospective randomized trials are warranted [2].

Despite those encouraging results for disease control, the higher radiation energy of Actinium-225 is associated with an increased risk of renal and hematological toxicity when compared with β⁻-emitting Lutetium-177 [2]. In patients scheduled for alpha therapy, renal impairment occurs in approximately 42% of patients [2], especially in those with prior [^177^Lu]Lu-PSMA RLT failure [5]. Similarly, grade 3 anemia has been observed in about one-third of pretreated subjects [5] and thus, identifying reliable predictors of such side effects is essential for optimizing patient selection and treatment safety.

Routine monitoring of laboratory markers reflecting renal and bone marrow function is recommended to mitigate these risks [6]. Additionally, Technetium-99m ([^99m^Tc]Tc)-mercaptoacetyltriglycine (MAG3) renal scintigraphy is routinely applied in those patients to assess urinary outflow obstruction and to quantify the tubular extraction rate (TER) [6]. However, the predictive value of MAG3 scintigraphy remains controversial, even under [^177^Lu]Lu-PSMA RLT [7]. For instance, it was demonstrated that under [^177^Lu]Lu-PSMA RLT blood-based estimated glomerular filtration rates outperforms TER in predicting renal toxicity [7]. Positron emission tomography/computed tomography (PET/CT) with PSMA-targeted tracers—commonly used to confirm PSMA expression before initiating RLT—may also provide valuable information on renal function [8]. For example, *Rassek* et al. demonstrated that renal uptake on PSMA PET/CT correlates with established functional markers such as TER [8]. PET-based renal functional imaging may offer important advantages over conventional renal scintigraphy, including the higher spatial resolution of PET and the ability to achieve absolute quantification using standardized uptake values [9]. Moreover, PSMA-targeted PET is already routinely performed in patients scheduled for RLT; therefore, these data could be readily leveraged for renal functional assessment [6]. The predictive value of PET-derived renal uptake for nephrotoxicity during [^225^Ac]Ac-PSMA RLT, however, remains unknown. Furthermore, PSMA-targeted PET-based quantification of tumor burden may help to identify patients at higher risk for hematological toxicity, particularly those with extensive skeletal metastases [10].

The aim of this study was to evaluate the predictive value of Fluorine-18 ([^18^F]F)-PSMA-1007 PET-derived renal SUV and tumor volume, in comparison with established renal function tests and laboratory markers, for renal and hematological adverse events during [^225^Ac]Ac-PSMA-I&T RLT.

## Materials and methods

### Patient population

We retrospectively included 26 patients (mean age: 72 ± 6 years) scheduled to receive [^225^Ac]Ac-PSMA-I&T RLT. All patients had progressive mCRPC despite multiple prior treatments, including androgen deprivation therapy, abiraterone acetate, enzalutamide, docetaxel, cabazitaxel, and [^177^Lu]Lu-PSMA RLT in most cases (Table 1). Hematotoxic and nephrotoxic previous therapies were as follows: [^177^Lu]Lu-PSMA RLT, olaparib and chemotherapy, e.g., docetaxel and cabazitaxel [11–14]. All patients demonstrated PSMA-avid disease on [^18^F]F-PSMA-1007 before commencing targeted alpha therapy. Exclusion criteria for therapy were preexisting severe bone marrow insufficiency and chronic kidney disease (common terminology criteria for adverse events (CTCAE) 5.0 ≥ grade 4 [15]. [^225^Ac]Ac-PSMA-I&T RLT was performed in accordance to the German Medicinal Products Act, AMG § 13.2b and the Declaration of Helsinki. Institutional review board approval was obtained (25–0130). Parts of this cohort were previously published in other analyses not focusing on toxicity and prediction of adverse events [16–18].

**Table 1:** Patient characteristics (*n* = 26)

Variable (mean ± SD)	
Age	73 ± 6
Heigh (m)	1.75 ± 0.08
Weigh (kg)	80 ± 15
Gleason score	8 ± 1
Previous treatments (n=)	
Radical prostatectomy	13
Primary radiation therapy	5
Androgen deprivation therapy	26
Enzalutamide	18
Abiraterone acetate	23
Previous chemotherapy	24
Docetaxel	23
Cabazitaxel	10
[^177^Lu]Lu-PSMA RLT	23
Cycles (n=)	5 ± 3
Interval to last potentially haemato- or nephrotoxic therapy* (days)	93 ± 53
Standard laboratory value	
Hb (g/dl)	9.85 ± 2.01
WBC (×10^3^/µL)	5.27 ± 1.55
Platelets (×10^3^/µL)	225 ± 86
Creatinine (mg/dL)	1 ± 0.2
eGFR (ml/min/1.73m^2^)	80 ± 16
AP (U/I)	387 ± 387
LDH (U/I)	442 ± 271
PSA (µg/L)	569 ± 639
Site of tumor lesions (%)	
Osseous	100
Lymph nodes	58
Hepatic	15
Prostate bed	35
Imaging based parameters	
[^99m^Tc]Tc-MAG3-derived TER (ml/min/1.73m^2^)	196 ± 51
[^18^F]F-PSMA-PET/CT-derived renal SUV	9.89 ± 4.39
[^18^F]F-PSMA-PET/CT-derived PSMA-TV (ml)	1025 ± 913
[^18^F]F-PSMA-PET/CT-derived osseous PSMA-TV (ml)	963 ± 622

*including: Lutetium-177 prostate-specific membrane antigen radioligand therapy ([^177^Lu]Lu-PSMA RLT), olaparib and chemotherapy. SD, standard deviation; Hb, hemoglobin; WBC, white blood cell count; eGFR, estimated glomerular filtration rate; AST, aspartate transaminase; ALT, alanine transaminase; AP, alkaline phosphatase LDH, lactate dehydrogenase; PSA, prostate-specific antigen; Tc, Technetium; MAG3, Mercaptoacetyltriglycin; TER, tubular extraction rate; F, Fluor; PET, positron-emission tomography; SUV, standardized uptake value; TV, tumor volume

### Assessment of PET-derived parameters

Pretherapeutic [^18^F]F-PSMA-1007 PET/CT was conducted in all patients administering a median of 232.5 MBq (232 ± 42.7 MBq) [^18^F]F-PSMA-1007. One hour post injection image acquisition included a helical CT (120 kV) and a PET scan from the vertex to mid-thigh (2.5 min per bed position; iterative reconstruction using TrueX [three iterations, 21 subsets] with Gaussian post-reconstruction smoothing [2 mm full width at half-maximum]), as previously described [17]. A commercial software (Affinity 4.0.2, Hermes Medical Solutions, Stockholm, Sweden) was used for image analysis.

PET-based renal uptake was determined following the protocol established by *Rassek* et al. [8]. Briefly, a volume of interest was placed carefully over each kidney. Renal uptake was then quantified including all voxels above 30% of the maximum standardized uptake value (SUV) of the referring kidney, enabling assessment of SUV_mean_ for each organ. For patient-based analysis, we used averaged SUV of both kidneys.

For assessing PET-based PSMA-TV, we followed a previously described approach [10, 19]. In this regard, we manually segmented all tumor lesions including all voxels above a SUV threshold of 4 [19]. Subsequently, we calculated total PSMA-TV, as well as osseous PSMA-TV for the prediction of hematological side effects [10].

### Assessment of pretherapeutic [^99m^Tc]Tc-MAG3-derived TER

Before commencing [^225^Ac]Ac-PSMA-I&T RLT each patient underwent renal scintigraphy with [^99m^Tc]Tc-MAG3. [^99m^Tc]Tc-MAG3 was manufactured using the Technescan MAG3™ preparation kit (Curium; Boston, MA, USA), following the manufactures instructions and the European Medicinal Agency-certified Summary of Product Characteristics. 30 min before starting the examination, patients received 500 ml 0.9% sodium chloride intravenously. Per patient, 96.7 ± 5.2 MBq of [^99m^Tc]Tc-MAG3 were injected. Imaging was performed using a low-energy, high-resolution collimator and a 64 × 64 matrix, with dynamic acquisition over 30 min immediately after injection, followed by two-minute static imaging post micturition. We placed regions of interest over the kidneys, thereby allowing quantification of renal uptake. To identify patients with clinically relevant obstruction, 10 mg furosemide were applied intravenously 15 min after the [^99m^Tc]Tc-MAG3 injection. Through a separate intravenous access blood samples were drawn 20 and 28 min following the [^99m^Tc]Tc-MAG3 injection [20]. Applying Bubeck‘s method, plasma activity was measured to calculate the TER [21].

### [^225^Ac]-PSMA-I&T RLT

[^225^Ac]-PSMA RLT was administered as previously described [17]. Briefly, as part of clinical routine care, patients received an intravenous dose of 7.52 ± 0.97 MBq [^225^Ac]Ac-PSMA-I&T per treatment cycle. To minimize off-target radiation exposure, a 2000 ml infusion of 0.9% sodium chloride was initiated 30 min prior to therapy [6]. Additionally, each patient was given 20 mg furosemide two hours post-treatment. Customarily, treatment was continued until progress or occurrence of relevant toxicities, resulting in a total of 3 ± 1 treatment cycles per patient, with each cycle routinely scheduled at 8-week intervals.

### Assessment of adverse events

Blood samples were obtained at baseline, 4 to 6 weeks after and at each cycle. Each blood collection included a serum-gel, lithium-heparin and di-potassium-ethylendiaminetetraacetic acid (EDTA) Monovette^®^ tubes (Sarstedt, Nürnbrecht, Germany). The estimated glomerular filtration rate (eGFR) was calculated using the CKD-Epidemiology Collaboration (EPI)-formula. All analysis followed manufacturers’ instructions, in-house procedure guidelines and standard quality assurance procedures. In addition, hemoglobin (Hb), white blood cell count (WBC), aspartate transaminase (AST), alanine transaminase (ALT), alkaline phosphatase (AP), lactate dehydrogenase (LDH) and prostate-specific antigen (PSA) were measured.

Renal events were defined using a recently proposed approach by the *Kidney Disease: Improving Global Outcomes (KDIGO) Chronic Kidney Disease (CKD) Work Group* [22]. Specifically, a decline in eGFR of ≥ 20% during [^225^Ac]Ac-PSMA-I&T RLT was considered as renal event. Hematological side effects were classified according to common terminology criteria for adverse events (CTCAE) 5.0 [15].

### Statistical analysis

Statistical analyses were conducted using GraphPadPrism 9 (GraphPad Software, San Diego, CA, USA) and SPSS Statistics 29 Inc. (IBM; Chicago, IL, USA). Simple linear regression was carried out to evaluate associations between the different renal functional parameters. Paired T-test assessed differences in baseline and nadir laboratory values during treatment. Optimal cut-off values for predicting renal events and grade ≥ 3 anemia were determined using receiver operating characteristics (ROC) and the Youden index [23]. Associations between imaging-based and clinical parameters and the risk of adverse events were evaluated using the chi-square test. In addition, Kaplan-Meier curves and log-ranks were applied to assess adverse-event free survival based on the identified cut-offs.

## Results

### Low [^18^F]F-PSMA-PET/CT-derived renal SUV is associated with renal toxicity under [^225^Ac]Ac-PSMA-I&T RLT

**Fig. 1:**
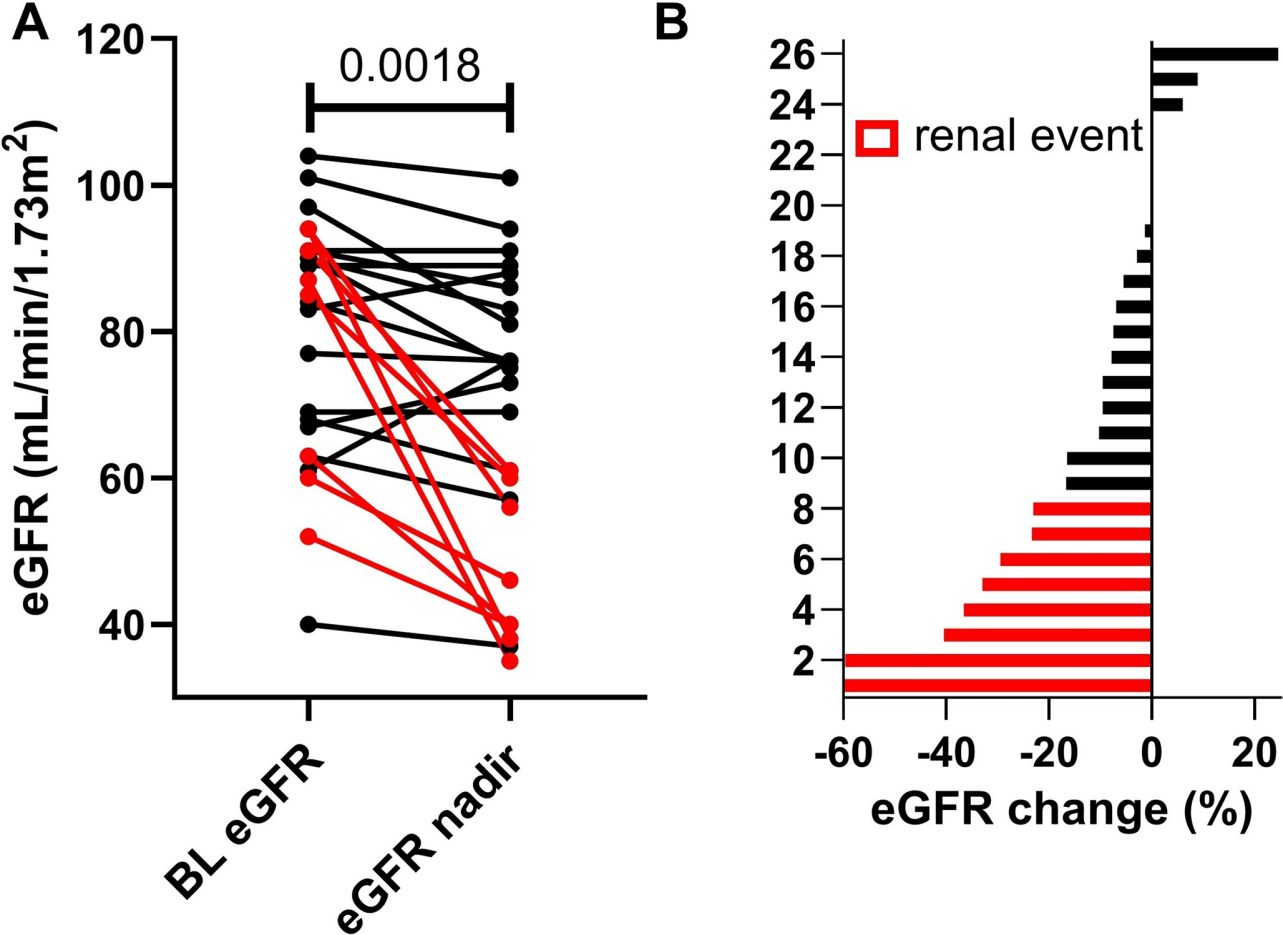
Renal Events under Actinium-225 prostate-specific membrane antigen radioligand therapy-I&T ([^225^Ac]Ac-PSMA-I&T RLT). (**A**) Line graph illustrating changes in estimated glomerular filtration rate from baseline to nadir. Each dot represents an individual patient with connecting lines indicating intraindividual change. Red lines highlight patients with a decline of ≥ 20%. (**B**) Waterfall plot depicting eGFR change (%), with patients ordered from strongest to lowest decline. Red bars highlight renal events, defined as ≥ 20% reduction in eGFR

[^225^Ac]Ac-PSMA-I&T RLT was associated with a significant decline in eGFR, from 80 ± 16 to 69 ± 20 mL/min/1.73m^2^ (*P* = 0.0018). Renal events occurred in 8 patients (Fig. 1).

There was a marked intraindividual variability in PET-derived renal uptake, with a mean renal SUV of 9.89 ± 4.39. Significant correlations were observed between [^18^F]F-PSMA-PET/CT-derived renal SUV and both eGFR (*r* = 0.498; *P* = 0.01) and the [^99m^Tc]Tc-MAG3-derived TER (*r* = 0.413; *P* = 0.036), validating our approach of measuring PET-derived renal uptake.

We performed ROC-analysis to determine optimal cut-off values to predict renal events (Table 2). Applying the identified cut-off values, [^18^F]F-PSMA-PET/CT-derived renal SUV (cut-off: 8.425) emerged as the strongest predictor for renal events with an odds ratio (OR) of 0.095 (95% confidence interval [CI], 0.014–0.668; *P* = 0.011; Table 3). A shorter interval between [^225^Ac]Ac-PSMA-I&T RLT and the last previously applied potentially nephrotoxic therapy was also linked to an increased risk for renal events (cut-off: 65.5 days; *P* = 0.046). In contrast, neither the baseline eGFR (cut-off: 65 mL/min/1.73m^2^; *P* = 0.245) nor the [^99m^Tc]Tc-MAG3-derived TER (cut-off: 178.5 mL/min/1.73m^2^; *P* = 0.165) reached significance.

**Table 2:** Receiver-Operating characteristics for renal events

Variable	AUC	Cut-off	*P*-value
Imaging based parameters			
[^18^F]F-PSMA-PET/CT-derived PSMA-TV (ml)	0.563	1670	0.584
[^18^F]F-PSMA-PET/CT-derived osseous PSMA-TV (ml)	0.528	1577	0.811
[^18^F]F-PSMA-PET/CT-derived renal SUV	0.715	8.425	0.053
[^99m^Tc]Tc-MAG3-derived TER (ml/min/1.73m^2^)	0.608	178.5	0.126
Clinical parameters			
Age	0.559	73	0.637
Number of [^225^Ac]Ac-PSMA-I&T RLT cycles received (n=)	0.642	2.5	0.255
Previous [^177^Lu]Lu-PSMA RLT cycles received (n=)	0.542	9	0.729
Interval to last potentially haemato- or nephrotoxic therapy* (days)	0.597	65.5	0.452
Hb (g/dl)	0.569	11.35	0.579
WBC (x10^3^/µl)	0.507	5.635	0.956
Platelets (x10^3^/µl)	0.549	188.5	0.697
eGFR (ml/min/1.73m^2^)	0.542	65	0.75
LDH (U/I)	0.597	411	0.437
AP (U/I)	0.549	476.5	0.697
PSA (µg/l)	0.535	150.5	0.781

*including: Lutetium-177 prostate-specific membrane antigen radioligand therapy ([^177^Lu]Lu-PSMA RLT), olaparib and chemotherapy. AUC, area under the curve; F, Fluor; PET, positron-emission tomography; TV, tumor volume; SUV, standardized uptake value; Tc, Technetium; MAG3, Mercaptoacetyltriglycin; TER, tubular extraction rate; Ac, Actinium; Hb, hemoglobin; WBC, white blood cell count; eGFR, estimated glomerular filtration rate; AST, aspartate transaminase; ALT, alanine transaminase; AP, alkaline phosphatase LDH, lactate dehydrogenase; PSA, prostate-specific antigen;

**Table 3:** Univariate predictors for renal events

Variable	Odds Ratio	95% CI	*P*-value
Imaging based parameters			
[^18^F]F-PSMA-1007 PET/CT-derived PSMA-TV (ml)			0.097
[^18^F]F-PSMA-1007 PET/CT-derived osseous PSMA-TV (ml)			0.097
[^18^F]F-PSMA-1007 PET/CT-derived renal SUV	0.095	0.014–0.668	***0.011***
[^99m^Tc]Tc-MAG3-derived TER	0.3	0.053–1.7	0.165
Clinical parameters			
Age	0.333	0.053–2.115	0.234
Number of [^225^Ac]Ac-PSMA-I&T RLT cycles received (n=)	2.6	0.462–14.63	0.272
Previous [^177^Lu]Lu-PSMA RLT cycles received (n=)			0.147
Interval to last potentially haemato- or nephrotoxic therapy* (days)	0.171	0.028–1.05	***0.046***
Hb	5	0.779–32.099	0.077
WBC	0.333	0.053–2.115	0.234
Platelets	4.455	0.447–44.413	0.178
eGFR	0.333	0.05–2.214	0.245
LDH	0.179	0.018–1.767	0.114
AP	3	0.452–19.928	0.245
PSA	4.455	0.447–44.413	0.178

Significant parameters are marked in bold and italic. *including: Lutetium-177 prostate-specific membrane antigen radioligand therapy ([^177^Lu]Lu-PSMA RLT), olaparib and chemotherapy. CI, confidence interval; F, Fluor; PET, positron-emission tomography; SUV, standardized uptake value; Tc, Technetium; MAG3, Mercaptoacetyltriglycin; TER, tubular extraction rate; Hb, hemoglobin; WBC, white blood cell count; eGFR, estimated glomerular filtration rate; AST, aspartate transaminase; ALT, alanine transaminase; AP, alkaline phosphatase LDH, lactate dehydrogenase; PSA, prostate-specific antigen. For PSMA-TV, osseous PSMA-TV and previous [^177^Lu]Lu-PSMA RLT cycles received Odds Ratio was not available due to a specificity of 100% of the ROC-derived cut-off

**Fig. 2:**
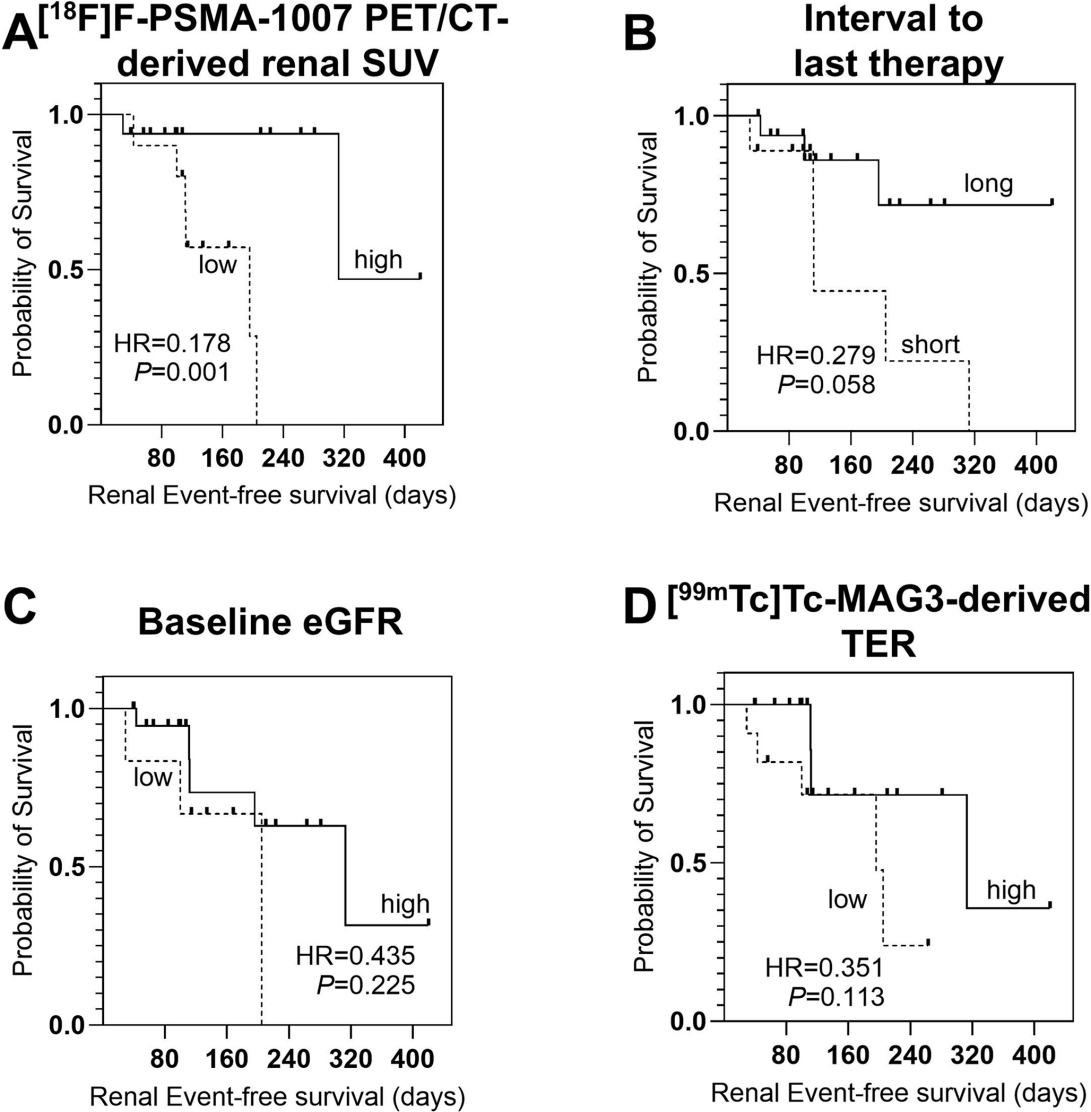
Kaplan-Meier Analysis for Renal Event-free Survival. Increased Fluorine-18 prostate-specific membrane antigen ([^18^F]F-PSMA) positron emission tomography/computed tomography (PET/CT)-derived renal standardized uptake value (SUV, **A**) was significantly associated with prolonged renal event-free survival PFS. There was tendency toward a shorter interval to potentially nephrotoxic systemic therapy including Lutetium-177 ([^177^Lu]Lu)-PSMA radioligand therapy, olaparib and chemotherapy, and subsequent shorter renal event-free survival, however, closely failing to reach significance (**B**) In contrast, estimated glomerular filtration rate (eGFR, **C**) and Technetium-99m ([^99m^Tc]Tc)-Mercaptoacetyltriglycine (MAG3)-derived tubular extraction rate (TER, **D**) were not predictive

Kaplan-Meier analysis (Fig. 2) revealed that patients with low [^18^F]F-PSMA-PET/CT-derived renal SUV experienced more frequent and earlier renal events, with a median renal event-free survival of 196 days compared to 313 days in those with higher SUV values (Hazard Ratio [HR], 0.178; *P* = 0.001). By contrast, the interval between [^225^Ac]Ac-PSMA-I&T RLT and the last previously applied potentially nephrotoxic systemic therapy (*P* = 0.058), baseline eGFR (*P* = 0.225) and [^99m^Tc]Tc-MAG3-derived TER (*P* = 0.113) did not significantly predict renal outcomes.

**Fig. 3:**
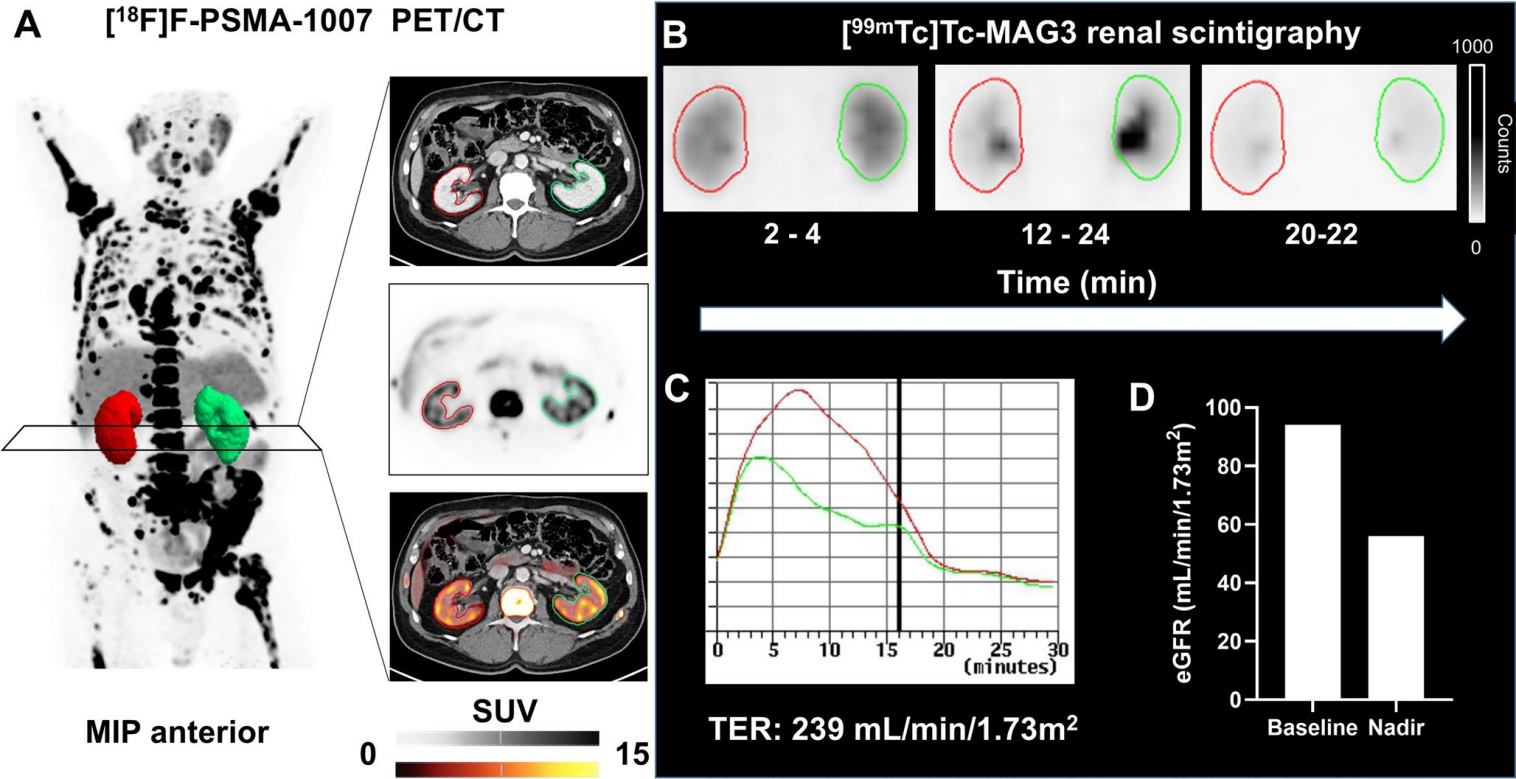
Case Example of a Patient with a Renal Event.(**A**) Fluorine-18 prostate-specific membrane antigen ([^18^F]F-PSMA) positron emission tomography/computed tomography (PET/CT) illustrating segmentation of the left (*in green*) and right kidney (*in red*) with maximum intensity-projection (MIP), CT, PET and fused PET/CT. Technetium-99m ([^99m^Tc]Tc)- Mercaptoacetyltriglycine (MAG3) renal scintigraphy with dorsal planar images (**B**, *left kidney marked in green*,* right kidney marked in red*) and the respective renograms (**C**, *green line for the left kidney*,* red line for the right kidney;* vertical line marking the administration of 10 mg furosemide) of the same patient. (**D**) illustrates estimated glomerular filtration rate (eGFR) at baseline and the nadir during Actinium-225 prostate-specific membrane antigen I&T radioligand therapy ([^225^Ac]Ac-PSMA-I&T RLT). This patient demonstrated renal standardized uptake value below the respective cut-off indicating higher risk for renal events. In contrast, tubular extraction rate and baseline eGFR suggested a lower risk. However, this patient experienced a 40% decline in eGFR, meeting the KDIGO CKD Working Grouping definition for renal event, thereby highlighting the predictive value of PET-based renal uptake

Figure 3 presents a representative case of a patient with low renal uptake on [^18^F]F-PSMA-PET/CT with a SUV under the identified cut-off, thereby indicating elevated risk for renal events. By contrast, both eGFR and [^99m^Tc]Tc-MAG3-derived TER indicated low risk due to respective values over the ROC-derived cut-off. However, this patient experienced a 40% decline in renal function during RLT, highlighting the superior predictive value of PET-derived renal uptake over conventional renal function metrics.

### Predictive value of clinical and imaging-based parameters for anemia under [^225^Ac]Ac-PSMA-I&T RLT

**Fig. 4:**
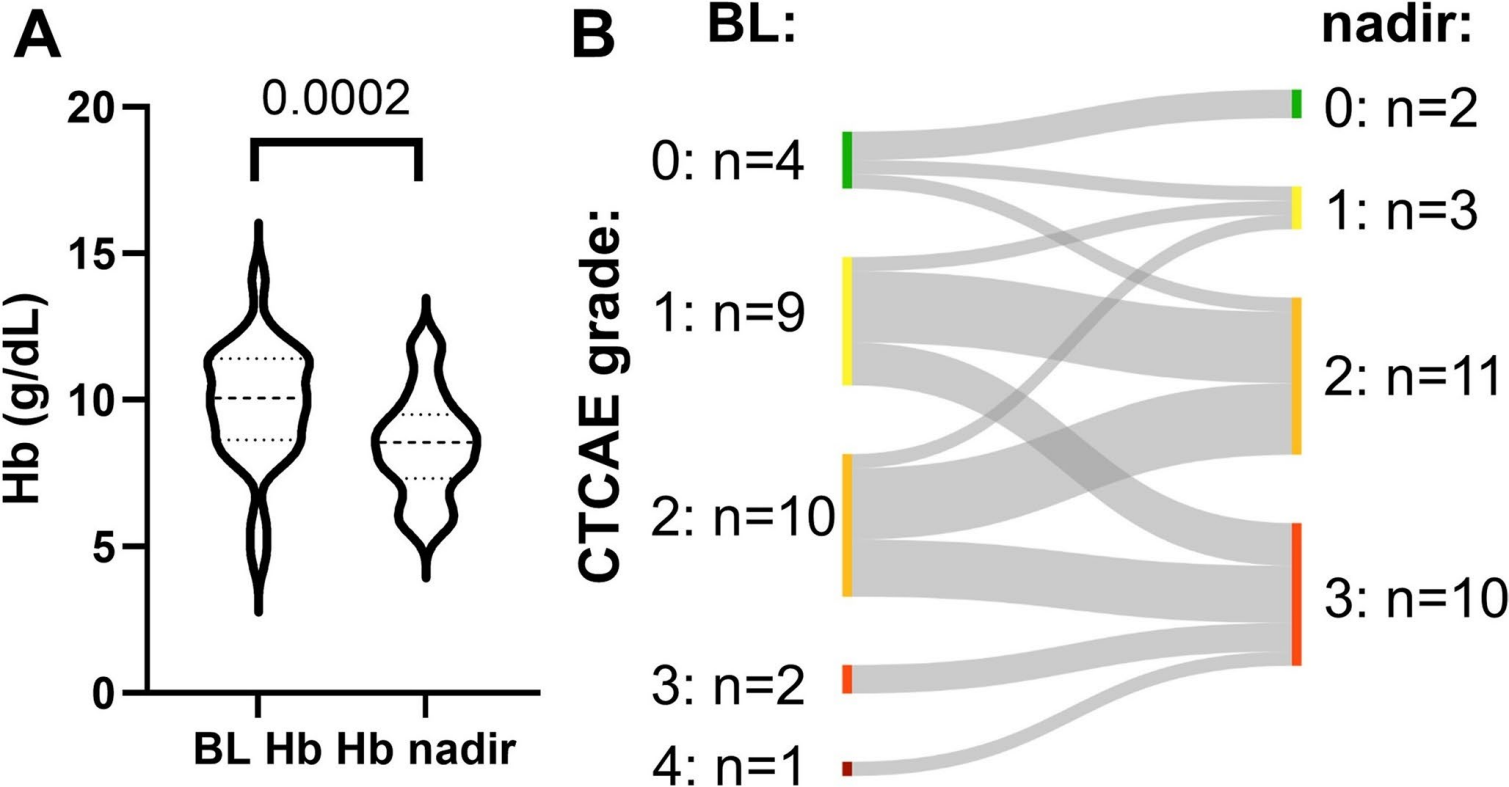
Anemia under Actinium-225 prostate-specific membrane antigen-I&T radioligand therapy ([^225^Ac]Ac-PSMA RLT). Violin plot demonstrating baseline hemoglobin and corresponding nadir during treatment (**A**). Sankey graph depicting anemia severity, graded according to common terminology criteria of adverse events (CTCAE) at baseline and during [^225^Ac]Ac-PSMA RLT (**B**). Hb declined significantly under treatment (*P* = 0.0002). In line, the incidence of clinically relevant grade ≥ 3 anemia increased from 12% at baseline (*n* = 3) to 38% during [^225^Ac]Ac-PSMA RLT (*n* = 10)

Hemoglobin significantly declined from 9.85 ± 2.01 g/dl at baseline to 8.5 ± 1.75 g/dL during therapy (–14%; *P* = 0.0002). Notably, many patients had already initiated treatment with relatively low Hb levels, reflecting cumulative bone marrow compromise from prior therapy lines. At baseline 3 patients (12%) had grade ≥ 3 anemia, which increased to 10 patients (38%) during [^225^Ac]Ac-PSMA-I&T RLT (Fig. 4).

ROC-analysis was conducted to identify optimal cut-off values for predicting grade ≥ 3 anemia (Table 4). Applying the identified cut-off values, baseline Hb (cut off: 8.35 g/dl) emerged as the strongest predictor for anemia (*P* = 0.002, Table 5), followed by platelet count (cut off: 178 × 10^3^/µL; *P* = 0.003), WBC (cut-off: 5.78 × 10^3^/µL; *P* = 0.018), AP (cut-off: 371 U/L; *P* = 0.031), osseous PSMA-TV (cut-off: 1396.83 mL; *P* = 0.031) and LDH (cut-off: 289.5 U/L; *P* = 0.037).

**Table 4:** Receiver-Operating characteristics for anemia

Variable	AUC	Cut-off	*P*-value
Imaging based parameters			
[^18^F]F-PSMA-1007 PET/CT-derived PSMA-TV (ml)	0.719	1023.78	***0.035***
[^18^F]F-PSMA-1007 PET/CT-derived osseous PSMA-TV (ml)	0.7	1396.83	0.061
[^18^F]F-PSMA-1007 PET/CT-derived renal SUV	0.619	8.83	0.322
Clinical parameters			
Age	0.559	67	0.642
Number of [^225^Ac]Ac-PSMA-I&T RLT cycles received (n=)	0.609	1.5	0.356
Previous [^177^Lu]Lu-PSMA RLT cycles received (n=)	0.55	3.5	0.672
Interval to last potentially haemato- or nephrotoxic therapy* (days)	0.516	65.5	0.896
Hb (g/dl)	0.756	8.35	***0.011***
WBC (x10^3^/µl)	0.794	5.78	***0.013***
Platelets (x10^3^/µl)	0.762	178	***0.027***
eGFR (ml/min/1.73m^2^)	0.5	60.5	1
LDH (U/I)	0.688	289.5	0.114
AP (U/I)	0.709	371	0.077
PSA (µg/l)	0.575	917.5	0.527

Significant parameters are marked in bold and italic. *including: Lutetium-177 prostate-specific membrane antigen radioligand therapy ([^177^Lu]Lu-PSMA RLT), olaparib and chemotherapy. AUC, area under the curve; F, Fluor; PSMA, prostate-specific membrane antigen; PET, positron-emission tomography; CT, computer tomography; TV, tumor volume; SUV, standardized uptake value; Hb, hemoglobin; WBC, white blood cell count; eGFR, estimated glomerular filtration rate; AST, aspartate transaminase; ALT, alanine transaminase; AP, alkaline phosphatase LDH, lactate dehydrogenase; PSA, prostate-specific antigen

**Table 5:** Univariate predictors for anemia

Variable	Odds Ratio	95% CI	*P*-value
Imaging based parameters			
[^18^F]F-PSMA-1007 PET/CT-derived PSMA-TV	5.133	0.922–28.57	0.054
[^18^F]F-PSMA-1007 PET/CT-derived osseous PSMA-TV	6.5	1.094–38.633	***0.031***
[^18^F]F-PSMA-1007 PET/CT-derived renal SUV	3.889	0.718–21.061	0.107
Clinical parameters			
Age	0.156	0.014–1.775	0.102
Number of [^225^Ac]Ac-PSMA-I&T RLT cycles received (n=)	0.156	0.014–1.775	0.102
Previous [^177^Lu]Lu-PSMA RLT cycles received (n=)	1.815	0.34–9.687	0.483
Interval to last potentially haemato- or nephrotoxic therapy* (days)	3.111	0.495–19.541	0.216
Hb			***0.002***
WBC	0.086	0.009–0.853	***0.018***
Platelets	0.044	0.004–0.484	***0.003***
eGFR			0.145
LDH	9	0.914–88.575	***0.037***
AP	6.5	1.094–38.633	***0.031***
PSA	6.429	0.563–73.351	0.102

Significant parameters are marked in bold and italic. *including: Lutetium-177 prostate-specific membrane antigen radioligand therapy ([^177^Lu]Lu-PSMA RLT), olaparib and chemotherapy. CI, Confidence interval; F, Fluor; prostate-specific membrane antigen; PET, positron-emission tomography; CT, computer tomography; TV, tumor volume; SUV, standardized uptake value; Hb, hemoglobin; WBC, white blood cell count; eGFR, estimated glomerular filtration rate; AST, aspartate transaminase; ALT, alanine transaminase; AP, alkaline phosphatase LDH, lactate dehydrogenase; PSA, prostate-specific antigen. For Hb and eGFR Odds Ratios were not available due to a specificity of 100% of the ROC-derived cut-off

**Fig. 5:**
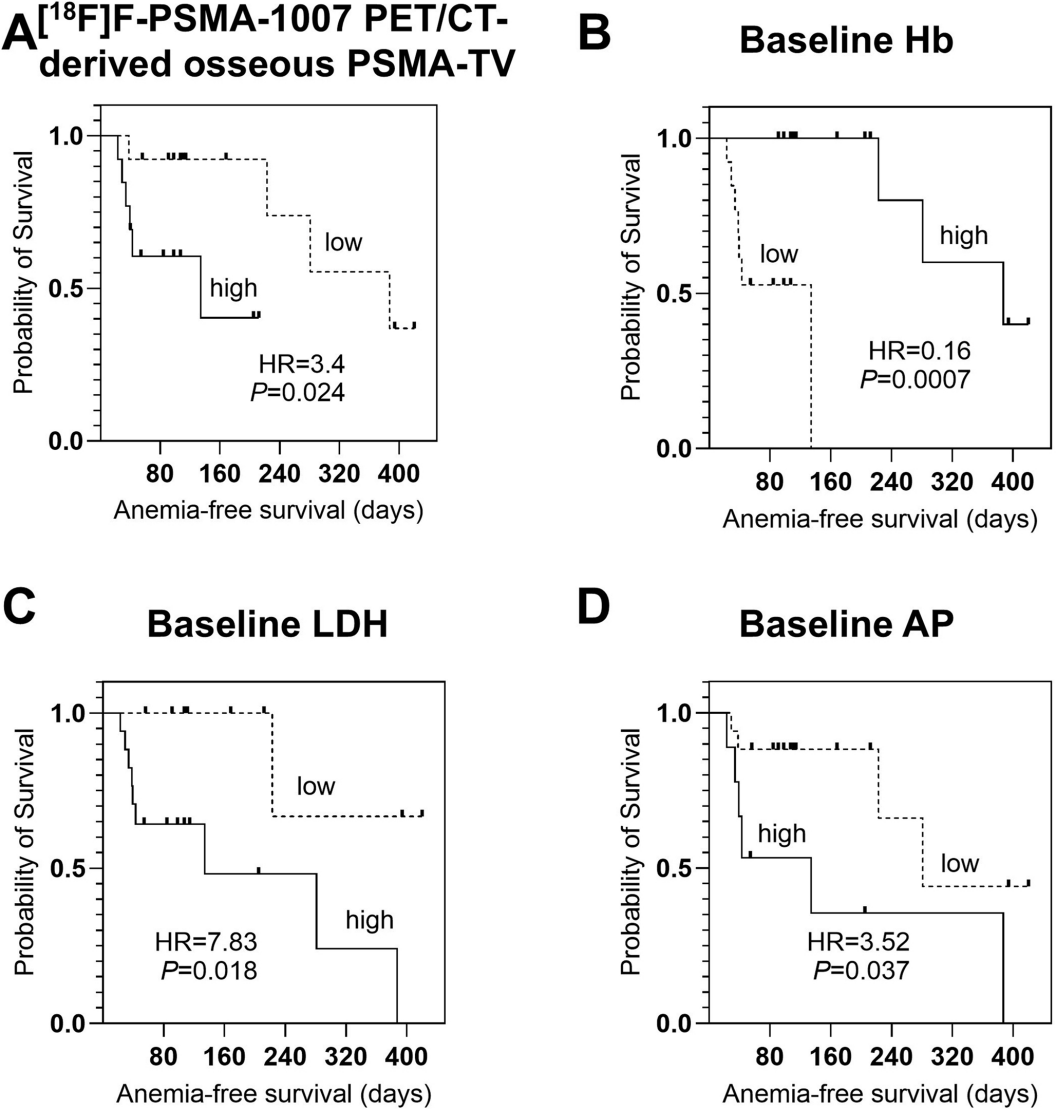
Kaplan-Meier Analysis for Anemia-free survival. Lower osseous prostate-specific membrane antigen-tumor volume (PSMA-TV, **A**) on Fluorine-18 ([^18^F]F)-PSMA positron emission tomography/computed tomography (PET/CT) was significantly associated with longer anemia-free survival, although baseline hemoglobin (Hb, **B**) proved to be a stronger predictor. Additionally, elevated Lactate dehydrogenase (LDH, **C**) and alkaline phosphatase (AP, **D**) were also significantly associated with anemia risk

Kaplan-Meier analysis (Fig. 5) revealed that patients with low baseline Hb levels experienced significantly more frequent and earlier onset of anemia, with a median anemia-free survival of 134 days compared to 387 days in patients with higher baseline Hb (HR, 0.16; *P* = 0.0007). Other significant predictors included elevated LDH (HR, 7.83; *P* = 0.018), increased ^18^F-PSMA-PET/CT-derived osseous PSMA-TV (HR, 3.4; *P* = 0.024) and high AP (HR, 3.52; *P* = 0.037).

### Other toxicities under [^225^Ac]Ac-PSMA-I&T RLT

With respect to hematologic parameters other than hemoglobin, we observed significant reduction in both WBC (from 5.27 ± 1.55 × 10^3^/µL at baseline to 4.01 ± 1.47 × 10^3^/µL during therapy [–24%; *P* < 0.0001]) and platelets (from 225 ± 86 × 10^3^/µL at baseline to 163 ± 72 × 10^3^/µL during therapy [–28%; *P* = 0.0005]). Nonetheless, clinically relevant cases of grade ≥ 3 leukopenia (*n* = 1; 4%) or thrombocytopenia (*n* = 2; 8%) were rare.

## Discussion

This study suggests that pretherapeutic [^18^F]F-PSMA-1007 PET/CT may offer useful predictive insights into the occurrence of adverse events during [^225^Ac]Ac-PSMA-I&T RLT. Specifically, renal SUV correlated with standard renal function markers (eGFR, TER) and was the strongest predictor of renal events. Similarly, baseline Hb identified patients at risk for severe anemia. These findings suggest that easily obtainable imaging and laboratory markers can support individualized risk stratification in this vulnerable patient population.

We observed grade 3 anemia in 38% of patients —higher than the 11% reported by *Ninatti* et al. [2], likely due to our more heavily pretreated cohort. A recent study limited to post-[^177^Lu]Lu-PSMA RLT patients, reported 35% grade 3–4 anemia, consistent with our results. Of note, treatment response rates are also lower in patients which have already experienced various previous treatment lines [3]. This underscores the importance of individualized toxicity risk assessment, particularly in patients with extensive prior therapies and poorer bone marrow reserves.

Patients in this study exhibited extensive skeletal tumor burden, with a median osseous PSMA-TV of 891 ml (IQR, 380–1506 ml). As expected, high tumor burden in the skeleton correlated with hematologic toxicity risk, consistent with prior [^177^Lu]Lu-PSMA RLT studies [24]. However, baseline Hb outperformed osseous PSMA-TV as a predictor, providing a simple and accessible risk marker. Further improvements in bone marrow reserve assessment including [^99m^Tc]Tc-antigranulocyte scintigraphy [25] may refine predictive models.

Applying recently published recommendations of the KDIGO CKD working group [22] we observed renal events in 31% of our enrolled patients, which is in line with previous studies reporting on renal functional decline during [^225^Ac]Ac-PSMA RLT [2, 26]. In our analysis, [^18^F]F-PSMA-1007 PET/CT-derived renal SUV emerged as the strongest predictor of renal events (OR, 0.095; *P* = 0.011) and was associated with shorter renal event-free survival. Of note, a shorter interval between [^225^Ac]Ac-PSMA-I&T RLT and the last previously applied potentially nephrotoxic systemic therapy was also associated with an increased risk for renal events (OR, 0.171; *P* = 0.046). This may indicate a reduced renal functional reserve in patients with a shorter therapy-free interval.

The use of PSMA-PET for renal functional imaging remains controversial due to potential confounding from urinary excretion [27] and thus, standardized assessment approaches are essential for reproducibility [8]. We adopted the established PET methodology proposed by *Rassek* et al. [8], and our findings demonstrated consistent correlations between PET-derived renal uptake and standard measures of renal function (eGFR, TER), supporting this approach as a reliable tool for PET-based kidney assessment. However, this approach does not enable to separate between PSMA-ligand accumulation due to PSMA-expression in the proximal tubules and renal excretion [28]. For PSMA-targeted RLT, it has been shown within the theranostic framework that increased PET uptake in tumor lesions is associated with higher tumor doses and improved treatment response [24, 29]. Consequently, one might expect that elevated renal SUV would similarly lead to higher renal radiation doses and, therefore, increased nephrotoxicity. In contrast, our data show a lower risk of renal impairment in patients with higher PET-derived renal SUV. This suggests that renal SUV likely reflects renal excretion and functional reserve rather than tubular PSMA expression [8]. In this context, dynamic PET imaging may offer improved renal functional assessment by distinguishing excretory processes from proximal tubular accumulation [30]. Finally, to enable more definitive conclusions, future investigations should focus on systematically correlating PET-derived measures of renal tracer uptake with detailed, kidney-specific dosimetric assessments obtained during radioligand therapy [31]. In addition, these imaging and dosimetric parameters should be longitudinally linked to subsequent changes in renal function over time. Such integrated analyses would help clarify the relationship between renal radiation exposure, functional imaging biomarkers, and clinically relevant renal outcomes, thereby strengthening the evidence base for risk stratification and toxicity prediction in this setting.

In this context, it is noteworthy that a higher number of administered [^225^Ac]Ac-PSMA-I&T RLT RLT cycles was not associated with an increased risk of renal adverse events. This observation is consistent with previous findings for [^177^Lu]Lu-PSMA RLT, where the cumulative number of treatment cycles was likewise not shown to correlate with renal toxicity [7, 32]. Topal et al. reported substantial interindividual variability in renal absorbed doses per cycle during [^177^Lu]Lu-PSMA RLT when dedicated dosimetric assessments were performed, with values ranging from 2.54 to 5.11 Gy [33]. Such variability may partly account for the limited predictive value of cycle number alone and further underscores the importance of post-therapeutic dosimetry to support personalized treatment planning and risk assessment.

Importantly, in this study, [^18^F]F-PSMA-1007 PET/CT outperformed [^99m^Tc]Tc-MAG3-derived TER for the predicting renal events, in line with reports questioning the predictive value of [^99m^Tc]Tc-MAG3-TER for CKD under [^177^Lu]Lu-PSMA RLT [7]. Since [^99m^Tc]Tc-diethylene triamine pentaacetic acid (DTPA) is freely filtered at the glomerulus, it may provide more accurate measurements of the renal function [34], future studies should explore whether [^99m^Tc]Tc-DTPA scintigraphy offers better predictive value in the [^225^Ac]Ac-PSMA RLT setting.

Moreover, our results underscore the advantages of PET-based renal imaging [9]. Although use of PSMA PET ligands might be more convenient as they are routinely used for tumor staging before RLT [6], dedicated PET radiotracers designed for renal function assessment may offer superior accuracy compared to ligands used off-label for this purpose [9]. One promising candidate is [^18^F]F-Fluorodeoxysorbitol (FDS) [35], which as a sorbitol derivative, is freely filtered at the glomerulus and provides sensitive imaging of glomerular function with inherent PET advantages over SPECT [35]. Preclinical studies in rats demonstrated excellent agreement between [^18^F]F-FDS PET and [^99m^Tc]Tc-DTPA scintigraphy [35], and early human studies have further supported its clinical utility [36]. Notably, next-generation total-body PET-scanners may further enhance the diagnostic performance of [^18^F]F-FDS PET/CT by enabling highly sensitive dynamic imaging with low activities [37].

PSMA is also physiologically expressed in salivary gland, leading to significant off-target accumulation of PSMA ligand in these tissues during PSMA-targeted RLT [28, 38]. As a result, the salivary glands are exposed to a considerable radiation, and xerostomia is a common adverse effect-even with less aggressive β^−^-emitter Lutetium-177-occuring in approximately 40% of patients under [^177^Lu]Lu-PSMA RLT [11]. This risk is further amplified with α-emitting therapies. For instance, *Feuerecker* et al. reported grade 1/2 xerostomia in all patients treated with [^225^Ac]Ac-PSMA-617, with 23% discontinuing treatment due to this side effect [5]. Unfortunately, xerostomia was not systematically assessed in our cohort, which precluded analysis of potential predictive factors. To address the risk of xerostomia, *Rosar* et al. proposed a tandem-therapy approach combining [^225^Ac]Ac-PSMA-617 with [^177^Lu]Lu-PSMA-617, aiming to reduce salivary gland toxicity [39]. In their study of 17 patients, only one patient developed new-onset xerostomia grade 1 during tandem-RLT [39]. Notably, other toxicities such as anemia or CKD were also infrequent in this cohort [39].

However, it is important to note that patients in that study received only a single cycle of tandem-RLT [39], and whether this approach offers sustained safety benefits over multiple cycles remains uncertain. Xerostomia, although frequently reported in patients undergoing [^225^Ac]Ac-PSMA RLT [5], was not systematically assessed in this cohort and therefore not available for analysis, thus leaving the herein presented safety data incomplete. Future studies should prospectively capture xerostomia and evaluate mitigation strategies such as tandem [^225^Ac]Ac/[^177^Lu]Lu-PSMA RLT over extended treatment courses also evaluating potential predictive markers for risk stratification.

Limitations of this study include the retrospective single-center design, small sample size and lack of external validation. Although key potential confounders for renal and hematologic toxicities—such as patient age and prior lines of treatment—were accounted for in the univariate analyses, the limited sample size precluded the performance of a robust multivariate analysis. Consequently, the findings presented here should be interpreted with caution, as they are exploratory in nature. Confirmation and refinement of these preliminary observations will require validation in larger, more comprehensive patient cohorts that allow for adequate adjustment of multiple covariates. Moreover, we herein only included [^18^F]F-PSMA-1007 PET/CT, future research should also investigate whether Gallium-68-labeled radiotracers yield similar predictive utility in this context. Finally, laboratory parameters for toxicity assessment were evaluated at baseline and again 4 to 6 weeks after each treatment cycle. Although this approach reflects a well-established and clinically validated follow-up protocol, particularly for the detection of clinically relevant and potentially therapy-limiting toxicities, a longer follow-up period would be preferable to more comprehensively capture delayed or long-term toxic effects.

## Conclusions

In this retrospective study, [^18^F]F-PSMA-1007 PET/CT-derived renal SUV and tumor volume were associated with renal and hematologic toxicity during [^225^Ac]Ac-PSMA-I&T RLT. Renal SUV was the strongest predictor for renal events, while baseline Hb provided superior predictive value for on-set of anemia. These findings support the use of PET imaging and routine laboratory markers for pre-treatment risk stratification, enabling more personalized management in this fragile population.

## Data Availability

The herein presented data is available in case of a reasonable request from the corresponding author.
